# Compact, Broadband, and High-Gain Four-Port MIMO Antenna for Future Millimeter Wave Applications

**DOI:** 10.3390/mi16050558

**Published:** 2025-05-03

**Authors:** Esraa Mousa Ali, Shine Let Gunamony, Mohamad A. Alawad, Turki Essa Alharbi

**Affiliations:** 1 Faculty of Engineering, Communications and Computer Engineering Department, Al-Ahliyya Amman University, Amman 19111, Jordan; e.ali@ammanu.edu.jo; 2 Department of ECE, Karunya Institute of Technology and Sciences, Coimbatore 641114, India; shinelet@gmail.com; 3 Department of Electrical Engineering, Imam Mohammad Ibn Saud Islamic University (IMSIU), Riyadh 11623, Saudi Arabia; 4 Department of Electrical Engineering, College of Engineering, Taif University, P.O. Box 11099, Taif 21944, Saudi Arabia; turki.alharbi@tu.edu.sa

**Keywords:** MIMO antenna, broadband wireless access, compact electronics, innovation systems, 5G, energy sustainable development, mm wave

## Abstract

A wideband antenna with a relatively compact size along with a multiple input and multiple output (MIMO) configuration for millimeter wave applications is proposed in this work. The antenna offers a low profile and simple structure. First of all, an antenna is designed using Rogers RT/duroid 6002 (Rogers Corporation, Chandler, AZ, USA) with a thickness of 0.79 mm, offering wideband ranges from 21 to 35 GHz. Subsequently, the unit element is converted into a four-port MIMO antenna to improve the capacity of the system, resulting in a high data rate, which is critical for 5G as well as for devices operating in the mm wave spectrum. The proposed work exhibits total dimensions of 24 × 24 mm^2^ and offers a peak gain of 8.5 dBi, with an efficiency of more than 80%. The MIMO performance parameters are also studied, and the antenna offers exceptional performance in terms of mutual coupling (S_ij_) without inserting a decoupling structure, envelop correlation coefficient (ECC), and diversity parameters. The proposed MIMO antenna offers a minimum isolation of −25 dBi and an ECC of less than 0.018. All the other MIMO parameter values lie below the acceptable range. The High Frequency Structure Simulator (HFSS) EM software (v.19) tool is used to analyze the antenna and study its performance. The simulated outcomes are verified by fabricating a prototype, where the result offers a good comparison among both results. Moreover, the contrast in terms of different performance parameters is carried out amongst recent research articles, highlighting the key contribution of the presented design. A compact size antenna with a wideband, simplified structure, and stable performance throughout the working band is achieved; thus, it is a solid contender for mm wave applications and 5G devices.

## 1. Introduction

Recently introduced and forthcoming 5G communication systems are promising in reshaping the wide range of daily routine work and industrial work by improving data transfer and reducing the latency rate. From mobile communication to wireless applications, as well as the concept of smart cities, smart vehicles, and Internet of Things (IoT), these varieties of systems are working or will start working using the mm wave spectrum [1,2,3]. Furthermore, with the expansion and evolution of 5G technology, work life will also change as more devices tend to shift toward the usage of wireless networks to work efficiently [4,5]. Thus, the mm wave spectrum is a possible solution to meet end user requirements, as the wideband and higher data transfers are only feasible by using the mm wave spectrum. Therefore, various frequency bands are allocated for 5G networks by the Federal Communications Commission (FCC), wherein the most popular are 24, 28, 37, 39, and 47 GHz [6,7]. These allocations of frequency band spectra are conducted by considering various variables that may affect the performance of wireless systems, and they have the tendency to improve the connectivity among devices by offering an enhanced data rate, which is critically important for smooth and stable performance [8,9,10].

In addition to their numerous applications and associated advantages, these frequency band spectra also suffer some setbacks, including poor non-line of sight (NLoS) communication due to low power, which consequently results in no passage through obstacles; thus, they are more effective for line of sight (LoS) communication systems [11,12]. Moreover, the high signal loss in harsh atmospheric conditions like rain and smog also limits their stable connectivity [13,14,15]. In addition to these issues, the future communication network demands a high-speed data rate to facilitate a big pool of people and exponentially increase the number of devices per capita. Therefore, mm wave systems require a wideband antenna with a high-gain capability, which improves the overall transmitted power of the system, resulting in long-range communication as compared to low-gain antennas. Thus, researchers put a lot of effort into designing various wideband, high-gain, and compact-size antennas [16,17,18,19]. A fractal printed antenna and its four-element array is presented in [16], achieving a high gain of 11 dBi at the cost of narrow bandwidth ranges from 27.45 to 28.86 GHz. Likewise, an integrated antenna system with two sets of array antennas working in the microwave and millimeter wave band spectrum is presented in [17]. Although this work offers a compact size, it has the setback of a narrow band and peak gain around 9.86 dBi, which is relatively low for array antennas. Contrary to these antennas, in [18,19], a metasurface is utilized to achieve a high gain of 11.4 dBi and 11.2 dBi, respectively. However, the overall size of the antenna is compromised along with the limitations in the bandwidth.

An antenna operating over a 5G millimeter wave band spectrum not only requires high gain with compact size, but also requires a simplified geometrical structure to avoid unnecessary fabrication errors and rapid mass production [20,21]. Consequently, it is critically important to design an antenna with the aforementioned parameters, along with an MIMO configuration with strong performance in terms of isolation and gain, to improve the signal propagation in a 5G communication network [22,23]. The application of frequency selective surface (FSS) technology is also used to improve the bandwidth, gain, and isolation of antennas; however, it results in a massive size, which limits its applications for compact devices [24]. In some cases, additional patches are loaded between MIMO elements to improve the performance of the antenna [25]. Although the technique is quite effective for mutual coupling reductions, it is only explored for narrowband MIMO antennas with an operational bandwidth of 718 MHz, ranging from 27.66 GHz to 28.378 GHz. Contrary to them, mutual coupling can also be reduced without introducing additional elements; for said purpose, the antennas are usually placed orthogonally or in an inverted position with reference to the unit element [26,27]. The mutual coupling of less than −15 dB is achieved with an overall size of 20 mm × 24 mm, yet the antenna suffers from the narrow bandwidth of 23.51–26.54 GHz [26]. In [27], the orthogonal placement results in a mutual coupling of less than −19 dB, with the highest value of gain of 9 dBi at the cost of an operational bandwidth of 27.76–28.4 GHz. The use of metamaterial directors is another effective technique to obtain an isolation of −20 dB in the whole operational bandwidth, yet the size and the bandwidth are the major setbacks of this work [28,29].

Thus, the aforementioned literary work shows that there is a huge room for smart antenna design offering features including, but not limited to, size compactness, wide operational bandwidth, and high gain. The conversion of the unit element of an antenna [30] into an MIMO antenna, and obtaining high-performance parameters without the insertion of any decoupling structure, EBGs or metamaterial layer, is also a key challenge. The selection of a suitable substrate material is another fundamental parameter of the antenna designing, as it is important for practical implementation and various applications of the antenna [31]. Moreover, the MIMO antenna should offer low coupling and ECC, while maintaining an electrically small size along with a simplified geometrical configuration. Meeting these features will show the potential of the antenna design for present and future 5G devices, as well as other applications that utilize the mm wave band spectrum. Therefore, in this paper, efforts have been put into designing an antenna operating on 28 GHz, while covering the globally allocated band spectrum, and having an overall smaller size, low profile, and simple geometrical configuration. The antenna offers strong performance parameters in standalone configuration as well as for the MIMO configuration by providing a wide operational band, high gain, and good value of isolation. Furthermore, the features below make the proposed antenna novel as compared to published works in the literature.

- Novel geometry with simple structure and compact size.
- Ease of fabrication and integration with other circuit components due to simple geometrical structure.
- Operates in wideband with high gain.
- Self-decoupled and offers low mutual coupling without the need of any additional decoupling structure.
- Offers high-performance MIMO parameters meeting the standard limits.

The remaining paper is divided into two major sections. In Section 2, the single element of the antenna as well as the MIMO antenna design is discussed. The design methodology and parametric analysis are briefly discussed to provide insight into the concept utilized. In Section 3, the antenna performance parameters are explained while comparing them with the measured results that are found using the fabricated hardware prototype. The MIMO antenna results along with a comparison with literary works are also discussed in this section. Finally, the proposed antenna system is described with references.

## 2. Proposed Antenna Design

### 2.1. Single Element of Antenna

**Figure 1:**
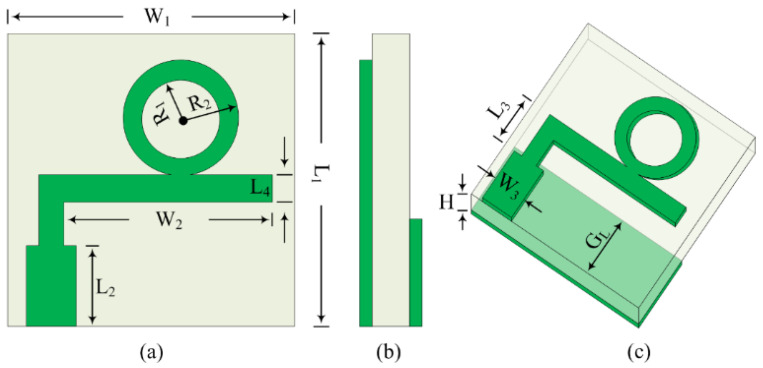
Geometry of millimeter wave antenna: (**a**) front view, (**b**) side view, (**c**) 3D view.

The structural configuration and geometry of the proposed simplified compact antenna designed for millimeter wave applications is provided in Figure 1. It is clearly depicted in the figure that the antenna structure contains an inverted L-shaped patch loaded with a circular ring-shaped stub [30]. A microstrip feedline with the left end is used to excite the radiating part of the antenna, whereas the antenna has a partial ground plane at the rear side of the substrate, which behaves as a reflector. A well-known and commercially available material, Rogers RT6002, with relative permittivity, loss tangent, and thickness of 2.94, 0.002, and 0.79 mm, respectively [31], is utilized to design the proposed antenna. The proposed antenna element has total measurements of L_1_ × W_1_ × H corresponding to 10 mm × 10 mm × 0.79 mm. An EM software tool, HFSS version 9, is utilized to configure, analyze, and investigate the performance parameters of the antenna while applying the proper boundary conditions. The proposed antenna’s optimized geometrical parameters are provided below.

L_1_ = 10; W_1_ = 10; L_2_ = 4; L_3_ = 2.5; L_4_ = 1.5; W_2_ = 8; W_3_ = 3; R_1_ = 1.5; R_2_ = 3; H = 0.79 (units in mm).

**Figure 2:**
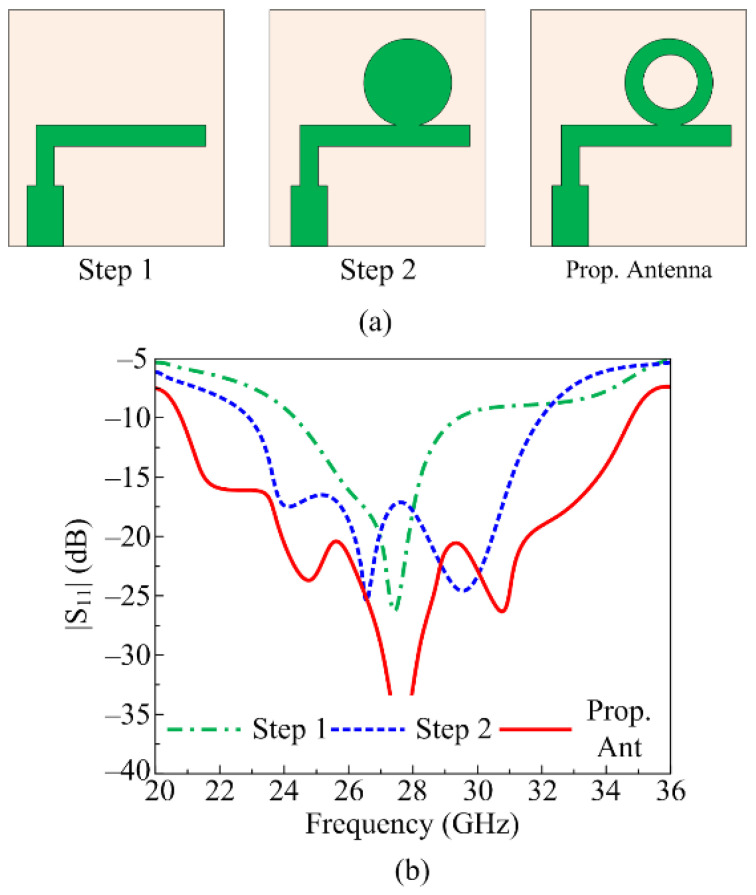
(**a**) Various stages followed to design the proposed dual-band antenna. (**b**) Impacts of various design steps on S_11_ plot.

Following various designing steps, the proposed UWB antenna with high gain is obtained. The design steps are converted in to three major stages, as follow:

*Stage 1.* In this design stage, an inverted L-shaped stub is loaded onto the microstrip feed line radiator, placed on top of the Roger 6002 substrate. The antenna contains partially ground planes. The designed antenna resonates at 28 GHz with a return loss value of around –18 dB, as given in Figure 2.

*Stage 2.* In this stage of designing the UWB antenna, a circular-shaped patch is placed on top side of the inverted L-shaped stub, such that they are connected with each other. The circular stub has a radius of R_2_ = 3 mm, as given in Figure 2a. This stage results in a minor shift of frequency along with a reasonable improvement in bandwidth. The stage offers a wideband of 23–32 GHz, as depicted in Figure 2b.

*Stage 3.* This is the final stage of designing the antenna, where a circular-shaped slot is etched from the circular stub loaded in the preceding step. As shown in Figure 2b, the antenna designed in stage 3 offers ultra-wideband ranges of 20.2–35.8 GHz with a minimum return loss value of −37 dB, as shown in Figure 2b.

During design stages, some parametric analyses are also performed, as discussed here, that affect the performance of the antenna. For optimized wideband design, the parametric analysis of two key design parameters is performed. The parametric analyses of the circular slot (R_1_) and the width of the feedline (W_3_) are carried out to show the impact of the performance of the S11.

**Figure 3:**
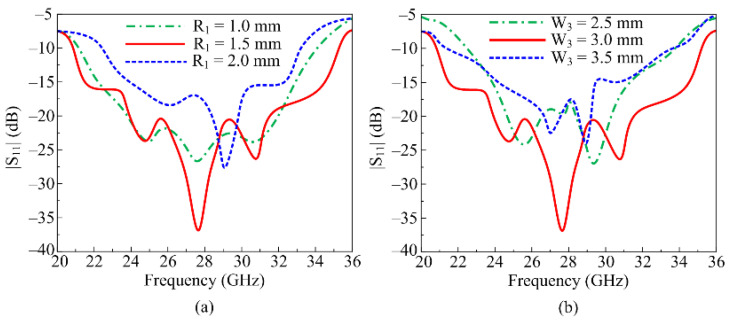
Parametric analysis of (**a**) radius of slot (R1) and (**b**) width of feedline (W3).

With R_1_ = 1.5 mm, the antenna covers a UW frequency range of 20.2–35.8 GHz, which is the optimal design of the proposed antenna. As shown in Figure 3a, the antenna bandwidth as well as the return loss is affected when the value is lowered to 1 mm. At this value, the antenna offers the bandwidth ranges 24–32 GHz, with a minimum return loss of −20 dB. On other hand, when the value is increased to 2 mm, the antenna operates from 24 GHz to 33 GHz, and offers a minimum return loss value of −23 dB, as depicted in Figure 3a. On the other hand, Figure 3b shows a parametric analysis of the feedline width (W3). The antenna bandwidth as well as the return loss suffers when the width is increased from the ideal value of 3 mm to 3.5 mm. The antenna offers the peak return loss of −24 dB and operates between 24.5 GHz and 31.25 GHz. The bandwidth along with return loss is also affected negatively when the feedline thickness is reduced to 2.5 mm, as depicted in Figure 3b.

**Figure 4:**
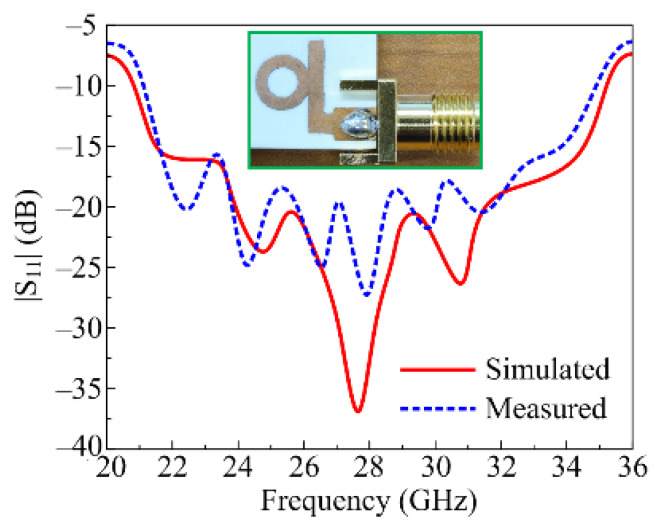
S parameter of suggested ultra-wideband antenna for millimeter wave applications.

A comparison among the tested and simulated scattering parameters (S_11_) of the ultra-wideband millimeter wave antenna is provided in Figure 4. It can be observed from the comparison that the antenna offers a bandwidth of 15.6 GHz, which covers the frequency range of 20.2–35.8 GHz. The proposed antenna offers an ultra-wide band along with low return loss in the operational region. The comparison in the figure also verifies that the measured results match well with simulated results, which validates the performance of the proposed antenna and its potential use in upcoming millimeter-wave compact devices operating over a wide band. Multiple resonances are obtained in the measured results as compared to the simulated results, which may be due to the fabrication tolerances, connector losses, and unforeseen parasitic effects resulting from the measurement setup.

**Figure 5:**
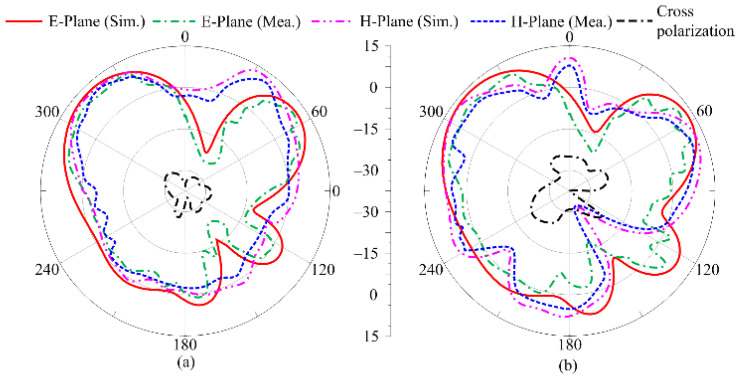
The radiation pattern of the recommended antenna at (**a**) 24.5 GHz and (**b**) 28 GHz.

Figure 5 presents the radiation patterns at selected resonant frequencies of 24.5 GHz and 28 GHz, and a comparison with the measured results. The antenna offers a broadside radiation pattern at both frequencies in the E plane, as shown in Figure 5. The radiation pattern at higher frequencies in the H plane is negligibly distorted and has a dumbbell shape. Slot-etching and side-feeding are the major factors resulting in slightly tilted radiation patterns. Since the overall radiation pattern achieved in the simulated results matches well with the measured results, showing the stability of the performance in terms of the radiation pattern, this is a potential candidate for 5G compact devices. 

**Figure 6:**
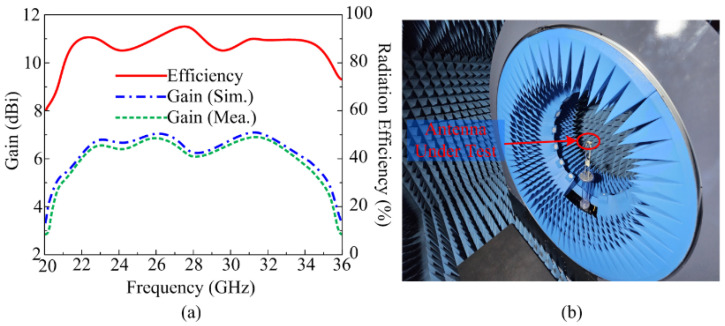
(**a**) Gain and efficiency of ultra-wideband antenna; (**b**) test for radiation parameters.

Figure 6a presents a comparison among the measured and simulated gain of proposed antenna along with the simulated radiation efficiency of the proposed antenna unit element. It is evident form Figure 6a that the antenna delivers a gain higher than 6 dBi throughout the operational region of 20.2–35.8 GHz. At resonance of 28 GHz, the antenna gain achieves its maximum value of 7.1 dBi, while at 30 GHz, it steps down to its minimum value of 6.2 dBi. The gain of the antenna is also validated after testing it inside the electromagnetic isolated chamber, as depicted in Figure 6b. Moreover, Figure 6a also shows the radiation efficiency of the proposed ultrawideband antenna. It is clear from the figure that the antenna has a high radiation efficiency of over 84% throughout the operational area. The radiation efficiency value is high and close to 93% at resonance frequencies of 24.5 GHz, 28 GHz, and 32 GHz. Thus, the proposed antenna becomes a suitable candidate for compact devices operating in the millimeter band spectrum, which require small-sized antenna with high gain and radiation efficiency.

### 2.2. Four Port MIMO Antenna

**Figure 7:**
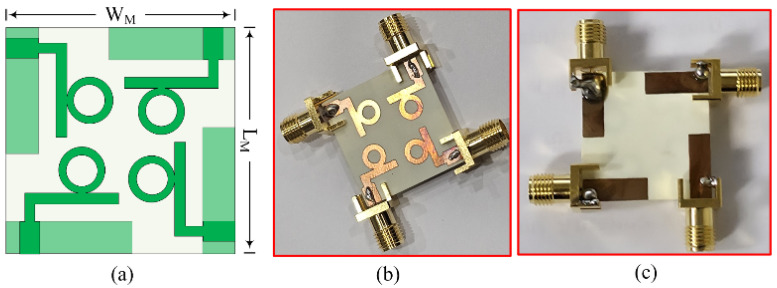
Geometrical configuration of the proposed MIMO antenna. (**a**) Layout, hardware prototype; (**b**) top view; (**c**) bottom view.

Figure 7a shows the structure of the MIMO antenna in the proposed work. It can be seen that the four-port MIMO configuration is derived from a single element with no changes in design parameters. Conventionally, filters are utilized to achieve filtering properties as well as low coupling and the control of the radiation patterns [32,33]. However, filters as well as the decoupling structure result in complex geometries, which make the fabrication process harder and increase the tolerance rate. The design parameters as well as the substrate material remain the same. The dimensions of the antenna increase due to the arrangement of four antenna elements. The four-port MIMO antenna has total dimensions of L_M_ × W_M_ × H = 24 mm × 24 mm × 0.79 mm. The gap between two opposite elements of the MIMO antenna is 3 mm, while the edge-to-edge difference between two consecutive elements is 2.2 mm. To validate the performance of the antenna, a hardware prototype is also fabricated, using Rogers RT/duroid 6002 as a substrate. The fabricated prototype is provided in Figure 7b,c.

## 3. Proposed MIMO Antenna Results

To verify the outcomes obtained from the software tool used to design the proposed antenna HFSS, a hardware prototype of the antenna is herein fabricated and analyzed. The performance parameters are measured in terms of the near field containing the reflection and transmission coefficient as well as the far field containing the gain, radiation pattern, and MIMO diversity parameters like ECC. The forthcoming part addresses the parameters of the antenna that can be used to analyze its performance, and which are discussed along with a comparison between the simulated and measured results.

### 3.1. Reflection and Transmission Coefficient

**Figure 8:**
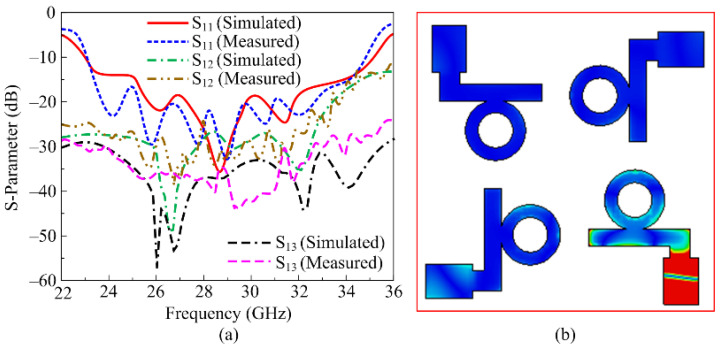
(**a**) Tested and predicted S-parameter; (**b**) current surface distribution at 28 GHz of the proposed MIMO antenna.

A comparison between the simulated and measured scattering parameters (reflection and transmission coefficient) is provided in Figure 8a. It can be seen that the antenna provides a wideband of 22.8–35.85 GHz with a minimum return loss of –35 dB at 28.5 GHz. The antenna also offers low mutual coupling up to –55 dB at 27 GHz, and a maximum value of −25 dB at 30 GHz. The proposed MIMO antenna offers self-decoupling, and so no additional structure is loaded to meet the standard value of less than -20 dB. Furthermore, self-decoupling enables the simple geometrical configuration that consequently mitigates the fabrication challenges. Due to the similarity in the results, the reflection coefficient of one element is provided in the figure, whereas the transmission coefficient following adjustments and of the opposite element is also given. Moreover, it is also clear from the results provided that there is strong agreement between the simulated and measured findings. The current distribution graphs of the proposed MIMO antenna are included in Figure 8b, wherein it can be seen that when either of the ports is excited, a very minute amount of current is introduced into the neighboring element, which consequently results in low coupling among the MIMO antenna’s elements.

### 3.2. Measured and Simulated Gain

**Figure 9:**
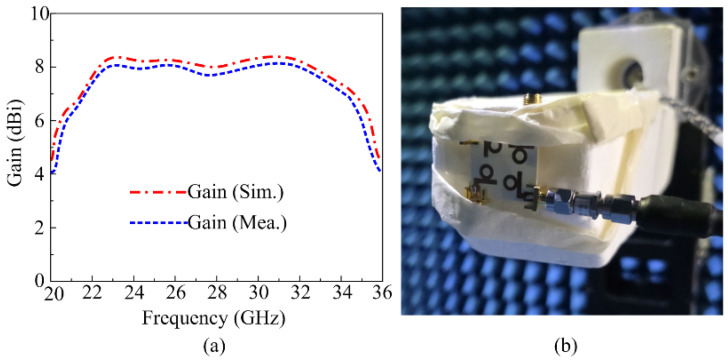
(**a**) Gain results of proposed antenna; (**b**) far-field measurement setup.

One of the most important far-field parameters of the antenna is its gain. In the operational bandwidth of 22–35 GHz, the proposed antenna provides a gain of higher than 7.55 dBi. Figure 9a shows that the gain reaches its maximum value of 8.3 dBi at 23 GHz, while it decreases to about 7.5 dBi between 26 and 30 GHz. Furthermore, there is good agreement between the measured results derived using chamber testing and the simulated results, as depicted in Figure 9. Owing to the high gain feature, the presented antenna possesses strong potential for use in upcoming high-gain devices that need a compact wideband antenna. Figure 9b shows a close-up shot of the proposed MIMO antenna in the chamber for far-field parameter testing.

### 3.3. Radiation Pattern

**Figure 10:**
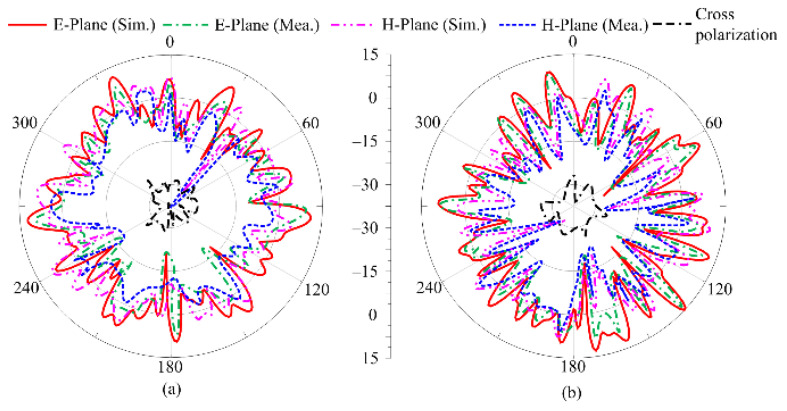
Tested and predicated radiation pattern of suggested ultra-wideband antenna at (**a**) 24 GHz and (**b**) 28 GHz.

A comparison of the simulated radiation pattern with the measured results is given in Figure 10. The radiation pattern is analyzed at the frequencies of 24.5 GHz and 28 GHz. It can be seen that the antenna offers a multibeam broadside-like radiation pattern at both frequencies. The pattern of the antenna is distorted, and multiple ripples are observed, which is due to the orthogonal placement and asymmetric structure of the antenna elements in the MIMO configuration, as well as the partial ground plane. The structure of the work also tilted the radiation pattern of the antenna; moreover, at both frequencies, the cross-polarization remained less than −27 dB, as depicted in Figure 10a,b. A strong agreement between the simulated and measured results, along with low cross-polarization, makes this a suitable candidate for diverse applications and multipath communication systems.

### 3.4. MIMO Parameters

**Figure 11:**
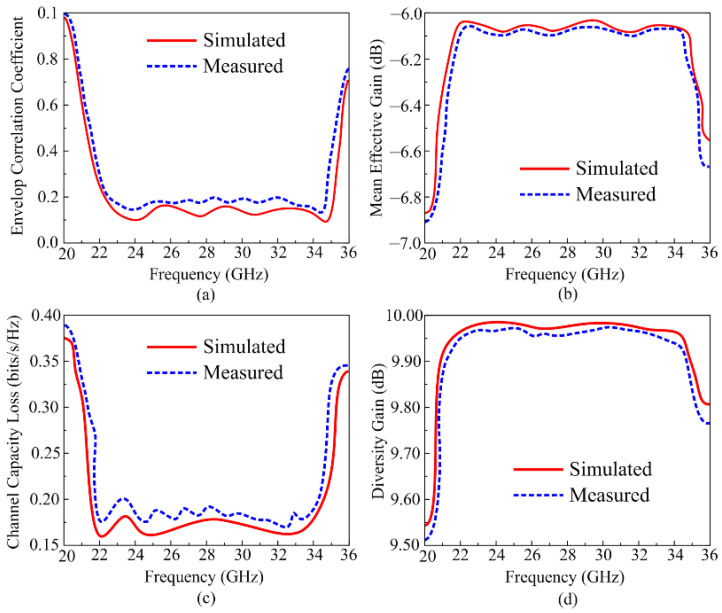
Tested and predicated MIMO parameters; (**a**) ECC, (**b**) MEG, (**c**) CCL, and (**d**) DG.

The performance of the MIMO antenna is herein studied by analyzing various parameters like envelop correlation coefficients (ECC), diversity gain (DG), mean effective gain (MEG), and channel capacity loss (CLL); the respective equations to calculate these parameters are discussed in [34,35]. For the proposed antenna these important parameters are analyzed by use of HFSS software and measured using the sample hardware prototype, as given in Figure 11a–d. Figure 11a shows the ECC results of the proposed antenna; ideally its value should be less than 0.2, and for the proposed antenna, the value of the ECC remains less than 0.18, which satisfies the practical application range. Furthermore, other parameters are also studied, and the simulated findings are compared with the measured results. MEG represents the received power in a fading environment, and for ideal cases its value should be less than –3 dB. The proposed work offers an MEG around –6.05 dB, which is acceptable. CCL in the MIMO system occurs due to correlation losses, and its value should be <0.5 bps/Hz/s in ideal cases. The proposed antenna offers CCL < 0.15 bps/Hz/s throughout the operational region, which is in an acceptable range for practical applications. Lastly, the DG represents losses that occur in transmission power, and its value should be 10 dB; however, considering losses in the practical environment, a value of more than 9.5 dB is acceptable, and the proposed antenna also satisfied that limit.

As is clear from Figure 11, the MIMO antenna offers good values for all important MIMO parameters, and these values stay below the acceptable range. The given figures additionally demonstrate that the results of the measured antenna and the simulated antenna are also in good agreement with each other. Furthermore, the proposed antenna is a good candidate for future wireless devices, owing to its advantage of low CCL and ECC and high DG. 

### 3.5. Literature Comparison

A brief performance comparison between the presented antenna and those in recently published works is given in Table 1. Antenna size, operating bandwidth, gain, mutual coupling, ECC, and CCL are compared. The antenna offers a compact size as compared to most of the related work, except for [16,26,27]. The work proposed in [16] offers narrow bandwidth, while the size is of the unit element, not the MIMO configuration. In [25], compact size is the result of mutual trade among the bandwidth and peak gain. Similarly, a compact-sized antenna with limited bandwidth and low value gain is achieved [26]. Lastly, the work proposed in [27] also suffers from narrow bandwidth and a bigger size as compared to the work proposed in this manuscript. Moreover, the work presented in [20] offers a similar-sized antenna with a slightly wider bandwidth; however, the gain of the antenna is quite low, at around 3.7 dB, which is not effective for a millimeter wave communication system. Thus, it is evident that the antenna provides strong performance in terms of standalone and MIMO configurations with wide band, high gain, and low profile. The results discussed above and comparisons with recent work indicate that the proposed antenna is a good option for applications in future compact devices.

## 4. Conclusions

A broad band, high gain, electrically undemanding, and low-profile MIMO antenna is suggested in this research article, targeting 5G applications. The antenna’s single element is made up of a ring-shaped patch loaded to an extended L-shaped feedline. A wide band and high gain are achieved by using three design stages consisting of L-shape stub optimization, the loading of the circular patch, and an etching slot along with the optimization of the whole antenna. Following that, a four-port MIMO antenna is constructed by reflecting the unit element in orthogonal placement targeting 5G applications that require multiple channels to achieve a high data rate transfer. Roger RT/duroid 5880 was used in the designing of the unit element, as well as the MIMO antenna. The MIMO antenna has an overall size of 24 mm × 24 mm × 0.254 mm. With a high gain of 11.5 dBi and a minimum isolation of −55 dB, the MIMO antenna provides wideband coverage from 22.8 GHz to 35.85 GHz. Additionally, the antenna offers strong MIMO parameter parameters that fall within the standard range. A hardware sample is utilized to verify the results produced by the HFSS electromagnetic software. It is evident from the results that both the unit element and the MIMO antenna offer a strong agreement in terms of the software-generated and measured results. Additionally, the proposed work’s performance is compared with recently published work in terms of low profile, operational bandwidth, gain, and other performance parameters of an MIMO antenna. The comparison table and antenna results indicate that the proposed antenna is the best fit for use in upcoming 5G mm wave devices requiring a compact antenna system with high performance parameters.

## Figures and Tables

**Table 1:** Comparison between the proposed antenna and recent published work.

Ref	Size (mm^3^)	Bandwidth (%)	Isolation (dB)	Gain (dBi)	Efficiency	ECC	CCL Bit/Hz/sec
[16]	12 × 32 × 1.575	5.03	–	10.12	80	–	–
[17]	110 × 75 × 0.508	17.5	–	10.29	90	–	–
[18]	24 × 24 × 1.574	20.71	–33	11.4	84	0.004 *	0.33
[19]	23 × 32 × 0.203	78	–28	11.21	–	0.004 *	–
[20]	24 × 24 × 0.203	71.4	–20	4.23	89	0.5	0.25
[22]	115 × 65 × 0.76	4.28	–30	9.65	80	0.16	–
[23]	32.4 × 32.8 × 0.8	12.86	–22	8.75	–	0.003 *	0.4
[24]	17.6 × 17.6 × 1.52	2.57	–25	8.75	–	–	–
[25]	24 × 20 × 1.85	11	–16	3.7	88	0.1	–
[26]	18 × 8.5 × 0.25	2.57	–20	7.73	88	0.03 *	0.15
[27]	31 × 48 × 0.254	17.9	–20	8.9	94	0.0015 *	–
[28]	45 × 45 × 0.25	24.64	–30	10	86	–	–
This work	24 × 24 × 0.79	46.6	–25	11.5	84	0.2	0.17

* Calculated using s-parameters.

## Data Availability

The original contributions presented in the study are included in the article, and further inquiries can be directed to the corresponding author.
